# Lipid Mediators From Timothy Grass Pollen Contribute to the Effector Phase of Allergy and Prime Dendritic Cells for Glycolipid Presentation

**DOI:** 10.3389/fimmu.2019.00974

**Published:** 2019-05-07

**Authors:** Nestor González Roldán, Regina Engel, Sylvia Düpow, Katharina Jakob, Frauke Koops, Zane Orinska, Claire Vigor, Camille Oger, Jean-Marie Galano, Thierry Durand, Uta Jappe, Katarzyna A. Duda

**Affiliations:** ^1^Junior Research Group of Allergobiochemistry, Airway Research North (ARCN), German Center for Lung Research (DZL), Borstel, Germany; ^2^Division of Experimental Pneumology, Research Center Borstel, Leibniz Lung Center, Airway Research Center North (ARCN), German Center for Lung Research (DZL), Borstel, Germany; ^3^Institut des Biomolécules Max Mousseron, IBMM, UMR 5247, CNRS, ENSCM, University of Montpellier, Montpellier, France; ^4^Division of Clinical and Molecular Allergology, Research Center Borstel, Leibniz Lung Center, Airway Research Center North (ARCN), German Center for Lung Research (DZL), Borstel, Germany; ^5^Interdisciplinary Allergy Outpatient Clinic, Department of Pneumology, University of Lübeck, Lübeck, Germany

**Keywords:** pollen, timothy grass, phytoprostanes, phytofuranes, CD1d molecule, allergic airway inflammation

## Abstract

Plant pollen are an important source of antigens that evoke allergic responses. Protein antigens have been the focus of studies aiming to elucidate the mechanisms responsible for allergic reactions to pollen. However, proteins are not the sole active agent present in pollen. It is known that pollen grains contain lipids essential for its reproduction and bioactive lipid mediators. These small molecular compounds are co-delivered with the allergens and hence have the potential to modulate the immune response of subjects by activating their innate immune cells. Previous reports showed that pollen associated lipid mediators exhibited neutrophil- and eosinophil-chemotactic activity and induced polarization of dendritic cells (DCs) toward a Th2-inducing phenotype. In our study we performed chemical analyses of the pollen associated lipids, that are rapidly released upon hydration. As main components we have identified different types of phytoprostanes (PhytoPs), and for the first time phytofurans (PhytoFs), with predominating 16-F_1t_-PhytoPs (PPF_1_-I), 9-F_1t_-PhytoPs (PPF_1_-II), 16-E_1t_-PhytoPs (PPE_1_-I) and 9-D_1t_-PhytoPs (PPE_1_-II), and 16(*RS*)-9-*epi*-ST-Δ^14^-10-PhytoFs. Interestingly 16-E_1t_-PhytoP and 9-D_1t_-PhytoPs were found to be bound to glycerol. Lipid-containing samples (aqueous pollen extract, APE) induced murine mast cell chemotaxis and IL-6 release, and enhanced their IgE-dependent degranulation, demonstrating a role for these lipids in the immediate effector phase of allergic inflammation. Noteworthy, mast cell degranulation seems to be dependent on glycerol-bound, but not free phytoprostanes. On murine dendritic cells, APE selectively induced the upregulation of CD1d, likely preparing lipid-antigen presentation to iNKT cells. Our report contributes to the understanding of the activity of lipid mediators in the immediate effector phase of allergic reactions but identifies a yet undescribed pathway for the recognition of pollen-derived glycolipids by iNKT cells.

## Introduction

Allergic diseases consist of several clinical conditions caused by hypersensitivity of the immune system to environmental factors that are normally harmless for most people. The prevalence of allergic diseases worldwide is rising dramatically in both developed and developing countries. This increase is especially problematic in children, who are bearing the greatest burden of the rising trend which has occurred over the last two decades (1). Grass pollen are, along with house dust mite, the most common inhalant allergen sources responsible for IgE-mediated allergies (2). An allergen is a protein, often glycosylated, that is presented by dendritic cells and together with additional factors leads to Th2 polarization and production of IgE antibodies (3). However, allergens are not delivered to the subject as pure proteins but as particles composed of various chemically different molecules in addition to the allergenic protein (4). The pollenkitt, an adhesive material present on the external layer of pollen grains, is mainly composed of lipids. These are responsible for reducing water loss and uptake, serve as reserve during pollen rehydration and activation of the stigma (5). Presently, the role of the lipids in the context of allergic inflammation is being increasingly discussed (6, 7). Lipids can be delivered alone, coming directly from the allergen source, as many different lipid species in different pollen samples with the ability to induce regulatory or stimulatory effects on the immune system, have been reported (8). They can either originate from microbes colonizing pollen (9) or be pollen-derived and transported bound to lipophilic allergens (6, 10).

Additionally, pollen grains contain lipid mediators that are released fast upon hydration, the so-called pollen associated lipid mediators (PALMs) (11). In the aqueous pollen extract (APE), containing PALMs hydroxy fatty acids-derivatives of linoleic acids (HODEs) (7) and phytoprostanes (PhytoPs) of class E_1_-, F_1_-, B_1_-, and A_1_ (12) were found. Birch and grass pollen activated and recruited polymorphonuclear granulocytes (PMNs) and eosinophils. This action was not fully mirrored by HODEs but rather by molecules structurally related to leukotrienes (LT). Cell activation was dependent on calcium mobilization and the upregulation of CD11b (7, 13). Additionally, PALMs were shown to elicit an effect on the activation and functional maturation of dendritic cells. In detail, birch pollen-APE selectively inhibited interleukin (IL)-12 p70 production of lipopolysaccharide (LPS)- or CD40L-activated DC, whereas IL-6, IL-10, and TNFα remained unchanged, leading to Th2 polarization. Interestingly this phenomenon was only dependent on PhytoP-E_1_ and not PhytoP-F_1_ or PhytoP-B_1_ (12). In *in vivo* studies in a murine sensitization model, however, neither PhytoP-E_1_ nor PhytoP-F_1_ evoked Th2 polarization by DCs (14).

PhytoPs are products of non-enzymatic oxidation of α-linolenic acid, the most abundant polyunsaturated fatty acids in plants (15–17). They were first described in 1998 by Mueller and Parchmann (18) and are known to be biomarkers of oxidative stress in plants (18). Two nomenclatures have been published (19, 20). Mueller and co-workers used Rokach nomenclature and named the phytoprostanes PP type I and type II depending on the abstraction of hydrogen radical on α-linolenic acid. The Taber nomenclature was approved by IUPAC (International Union of Pure and Applied Chemistry) and will be further used in this manuscript. To avoid any confusion, Mueller nomenclature will be mentioned in brackets. Further, Jahn et al. published in 2010 a cautionary note on the correct assignment of all series of PhytoPs (21). In addition, due to isomeric complexity and chemical instability analytical characterization of PhytoPs is challenging.

Similarly to the recognition of microbial lipids, pollen-derived lipids was mediated by CD1-restricted T cells, namely invariant Natural Killer T (iNKT) cells (22). iNKT cells are a major population of innate-like T lymphocytes expressing a semi-invariant T cell receptor composed of a canonical Vα14-Jα18 α chain with a variable Vβ8, −7, or−2 β chain in mice or Vα24-Jα18/Vβ11 in humans that is specialized for recognition of glycolipid ligands (23). One of their most important features is the ability to rapidly release a broad spectrum cytokines (24), thus they can modulate various immune responses, which is dependent on the structure of the agonist. The role of iNKT cells in the context of allergy is still under debate. Scanlon et al. demonstrated the recruitment of iNKT cells to the airways in the presence of airborne lipid antigen (25), furthermore the requirement of iNKT cells for the initiation of airway inflammation has been demonstrated (26). In contrast, other reports have postulated only a modulatory role of iNKT cells in asthma (25). However, a crucial prerequisite for iNKT cell activation is the expression and modulation of the glycolipid-presenting molecule CD1d on antigen presenting cells, such as dendritic cells.

In this work, we aimed to characterize the chemical composition of the fast-released lipid fraction from Timothy grass pollen and to investigate their potential contribution to the effector phase of allergy. We also evaluated whether the fast-released lipids can modulate the recognition of grass pollen particle-bound glycolipids through the up-regulation of the glycolipid-presenting molecule CD1d.

## Materials and Methods

### Preparation of Aqueous Pollen Extract (APE)

Pollen grains from Timothy grass (*Phleum pretense*) were purchased from Greer^®^ and kept at −20°C prior use. Aqueous pollen extract (APE) was obtained according to Traidl-Hoffmann et al. (7), with some modifications. For chemical analyses, 44 mg of grass pollen were extracted with 1 ml of water (concentration of 44 mg/ml) and 30 mg per ml of PBS (for immunological assays) for 30 min in ultrasonic bath followed by centrifugation (3,900 × g, at 20°C). The concentration of 30 mg/ml was used as it has been reported that 34 mg/ml of APE resulted in an average concentration of 3.9 × 10^10^ mol/L LTB_4_, which is known to be the concentration to induce migration in PMNs (7).

The supernatant containing fast-released lipids was sterile filtered (0.2 μm) and stored at −20°C prior to use.

In order to prepare protein free APE (APE_ProtK_), APE was treated with Proteinase K (Roche) for 4 h at 56°C.

### Enrichment and Fractionation of Lipids

To enrich fast-released lipids, PALMs, for a better detection in the analytical measurements, APE was further extracted with chloroform/methanol/water extraction (27) utilizing Branson Sonifier 250 for 20 min. on ice, Chloroform phase (APE_B/D_) containing PALMs was sterile filtrated (0.2 μm), dried and further fractionated on the silica gel 60 column (10 × 1 cm; 0.04–0.063 mm, Merck) with increasing volumes of methanol. Fractions 3, 4, and 5 (CHCl_3_/MeOH, 93/7, 90/10 and 80/20, v/v, respectively) were analyzed in detail utilizing GC/MS and LC/MS. Additionally, fraction 3 was further separated on reversed-phase Gilson 712 Gradient HPLC system equipped with Kromasil 100 C18, 5 μm, 250 × 10 mm (MZ-Analysentechnik GmbK) column with the following separation steps: isocratic 100% MeOH/H_2_O (1/1, v/v), 30 min; isocratic 100% MeOH 30 min.; gradient 100% MeOH – 100% CHCl_3_/MeOH (7/3, v/v) 30 min; isocratic 100% CHCl_3_/MeOH (7/3, v/v), 30 min. Flow rate was 2 ml/min, and the eluting material was detected with a light scattering Sedex 55 detector (Sedere).

### GC/MS Analyses

APE_B/D_ and its fractions were analyzed in GC/MS after different derivatizations methods. Samples were either hydrolyzed with 2m HCl/MeOH (1 h, 85°C), followed by a peracetylation (10 min., 85°C) or with 2m NaOH, followed by a methylation with trimethylsilyldiazomethane (30 min., 22°C) and silylation with *N*,*O*-Bis(trimethylsilyl)trifluoroacetamide (BSTFA) (3 h, 65°C). Additionally, to discriminate artifacts originating from acidic or alkaline hydrolysis APE_B/D_ was directly methylated (omitting the hydrolysis step) with trimethylsilyldiazomethane (30 min., 22°C) and silylated with BSTFA (3 h, 65°C).

GC-MS measurements were performed on Agilent Technologies 7890A gas chromatograph equipped with a dimethylpolysiloxane column (Agilent, HP Ultra 1; 12 m × 0.2 mm × 0.33 μm film thickness) and 5975C series MSD detector with electron impact ionization (EI) mode under autotune condition at 70 eV. The temperature programme was 70°C for 1.5 min, then 60°C min^−1^ to 110°C and 5°C min^−1^ to 320°C for 10 min.

### LC/MS Analyses

Phytoprostanes and phytofuranes profiling was performed using a micro-HPLC 200 plus (Eksingent Technologies, CA, USA) coupling with the tandem mass spectrometer Sciex QTRAP 5500 (Sciex Applied Biosystems, ON, Canada). Prior LC-MS injection, 100 mg of complete Timothy grass pollen was extracted with a Folch method according to the protocol of Yonny et al. (28) and Vigor et al. (29). The Timothy grass pollen extract (TGP), APE_B/D_ and originating from APE_B/D_ fractions 3, 4, and 5 underwent alkaline hydrolysis (1m KOH, 30 min., 40°C). Such obtained metabolites were concentrated by performing a solid phase extraction (SPE) step conducted on weak-anion exchange materials (Oasis MAX; 3 mL, 60 mg from Waters; Milford, MA, USA). Therewith metabolites were analyzed by micro-LC-MS/MS. The chromatographic separation of the phytoprostanoids was performed on a HALO C_18_ analytical column (100 × 0.5 mm, 2.7 μm particle size; Eksingent Technologies, CA, USA) held at 40°C and achieved by a gradient elution with 0.1% aqueous formic acid (A) and acetonitrile: methanol (80/20 v/v, B) with 0.1% additional formic acid. The gradient mode, delivered at 0.0 3mL.min-1, was started with 17% solvent B held for 1.6 min, increased to 21% solvent B at 2.85 min, to 25% at 7.27 min, to 28.4% at 8.8 min, to 33.1% at 9.62 min, to 33.3% at 10.95 min, and to 40% at 15 min. A maximum of 90% solvent B was reached at 16.47 min, and then returned to the initial conditions at 19 min. The Sciex QTRAP 5500 mass spectrometer detector operated in electrospray negative ionization mode. MS detection was performed by MS/MS using the MRM acquisition mode in a scheduled mode with an opening window of detection of ± 1 min (2 min in total) for the expected RT. Quantification of phytoprostanoids was achieved by the ratio between the peak area of each analyte and that of the corresponding IS. Data processing was achieved using MultiQuant 3.0 software (Sciex Applied Biosystems).

### Mice

*Cd1d*^−/−^ mice on a C57BL/6 background were kindly provided by Prof. Gisa Tiegs (UKE, Hamburg) and C57BL/6 controls were bred and housed at the Animal Care Facility of the Research Center Borstel. Mouse care and removal of organs was performed in accordance with institutional (RCB) guidelines.

### ESI MS

Electrospray Ionization Mass Spectrometry (ESI MS) of APE_B/D#3_ was performed in negative ion mode using an amaZon speed ETD—Instrument (Bruker Daltonics) equipped with ESI ion source. Sample was dissolved at a concentration of ~10 ng μL−1 in 10 mM ammonium acetate (50/50, v/v) mixture of chloroform and methanol and sprayed at a flow rate of 3 μL min−1. Capillary entrance voltage was set to 4.5 kV, and dry gas temperature to 180°C.

### Degranulation of the Murine Mast Cells, Chemotaxis, and IL-6 Production

Bone marrow-derived mast cells (BMMCs) were generated by cultivation of bone marrow cells from C57BL/6 mice in the presence of recombinant murine IL-3 and stem cell factor (SCF). Cells were maintained in complete medium consisting of 10% heat-inactivated fetal calf serum (Biochrom), 50 μM β-mercaptoethanol, 1 mM sodium pyruvate, 2 mM L-glutamine, nonessential amino acids, penicillin, streptomycin (all from Gibco), and for the first 2 weeks 10 μg/mL ciprofloxacin (Bayer) or Myco-3 (Applichem A5240) in Iscove's modified Dulbecco medium (IMDM) (Gibco) supplemented with 10 ng/mL IL-3 and 10 ng/ml SCF (both from R&D). After 5 weeks of culture, >95% of the total cells were BMMCs (CD117^+^ (c-Kit), FcεRIα^+^), T1/ST2^+^ and were negative for mycoplasma contamination.

Chemotaxis of BMMCs was measured using a transwell chamber system by assessing migration through a polycarbonate filter insert of 8-μm pore size in 24-well-plates (Corning Life Sciences). BMMCs were preincubated with complete IMDM and 1 ng/ml IL-3 overnight. SCF (10 ng/ml) or APE (1:10 dilution from the original 30 mg/ml extraction of pollen) in assay buffer [IMDM with 5% BSA (Sigma)] were loaded into the lower chambers in a volume of 600 μl. Cells were washed with assay buffer and added into the upper chamber (1 × 10^6^ cells/ml in 100 μl) and incubated at 37°C and 5% CO_2_ for 24 h. After incubation, cells from lower chambers were collected, washed in FACS-buffer (2% FCS, 0.1% NaN_3_, 0.2 mm EDTA in PBS) with 2 μg/ml PI (Sigma) and the total cell number was determined by flow cytometry after addition of AccuCheck Counting beads (Invitrogen). Dead cells (PI^+^) were excluded from analysis.

To induce degranulation or cytokine production 2 × 10^6^ cells/ml were cultivated in the presence of 200 ng/mL of dinitrophenyl (DNP)–specific IgE (clone SPE-7) (Sigma) over night at 37°C and 5% CO_2_. Cells were washed subsequently and stimulated either with PMA/Ionomycin (100 ng/mL, 100 nM, respectively), DNP-HSA as antigen (20 ng/mL, all from Sigma), APE, APE_B/D_, 16-F_1t_-PhytoP (PPF_1_-I), 9-F_1t_-PhytoP (PPF_1_-II), or 9-D_1t_-PhytoP (PPE_1_-II) (20 μg/ml), in round bottom 96-well-plates for 20 min at 37°C for degranulation measurement. For IL-6 production cells were cultivated for 24 h and culture supernatants were collected. Degranulation was measured by the detection of the lysosomal membrane protein CD107a (LAMP-1) translocation to the cell surface. Cells were stained with anti-mouse CD107a (clone 1D4B), CD117 (clone 2B8) and FcεRIα (clone MAR-1) (all from BioLegend) and washed with FACS-buffer containing 2 μg/ml PI. PI-negative cells were analyzed by flow cytometry.

To analyze IL-6 production, supernatants from stimulated cells were collected and IL-6 concentration was measured by specific enzyme-linked immunosorbent assay (ELISA) using specific antibodies and standard protein from R&D Systems.

### Maturation of Dendritic Cells

Bone marrow-derived dendritic cells (BMDCs) were generated by cultivation of bone marrow progenitor cells from C57BL/6 WT and *Cd1d*^−/−^ mice in complete medium consisting of 10% heat-inactivated fetal calf serum (Biochrom), 1% L-glutamine, 1% penicillin, 1% streptomycin, 0.1% Mercaptoethanol (all from Gibco) in RPMI (Gibco) supplemented with 200 ng/ml GM-CSF (PeproTech). After 7 days of culture, BMDCs were harvested.

For stimulation BMDCs (2 × 10^5^) from WT and *cd1d*^−/−^ were cultivated in the presence of either APE (1:10 dilution originating from 30 mg/ml of pollen) or 100 ng/ml of highly purified and lipopeptide-free *S. friedenau* LPS (kindly provided by Prof. Helmut Brade, Research Center Borstel, Germany) in round bottom 96-well-plates for 24 h at 37°C in 5% CO_2_. Maturation was assessed by the surface up-regulation of CD40, CD80, MHC class-II, and CD1d. Cells were labeled with anti-mouse CD80 (clone 16-10A1), CD1d (clone 1B1), CD40 (clone 3/23), CD11c (clone N418), MHC class-II (clone M5114.15.2) and washed in FACS-buffer containing 2 μg/ml PI. PI-negative cells were analyzed by flow cytometry.

### Flow Cytometry

Samples for flow cytometry were acquired either on a FACScalibur, a LSR-II (BD Biosciences) or a MACSQuant 10 (Miltenyi Biotec). The generated data were analyzed using the FlowJo cell analysis software (FlowJo, LLC).

### Statistical Analyses

Data are presented as mean values ± SD. Nonparametric one-way ANOVA and post tests were performed using GraphPad Prism version 5 software. A *p*-value of < 0.05 was considered as statistically significant.

## Results

### Lipid Mediators of APE Are Phytoprostanes and Phytofuranes

APE was obtained from complete pollen grains of Timothy grass with the yield of 30%. Since such water extract contains apart of fast-released lipids also proteins, lipids were further enriched utilizing chloroform/methanol extraction, yielding APE_B/D_ (0.44% to APE). Qualitative GC/MS analyses of APE_B/D_ revealed the presence of different PhytoPs depending on the used derivatization protocol. In the samples after solvolysis (acidic hydrolysis) we have detected 16-A_1t_-PhytoP (PPA_1_-I) and 9-J_1t_-PhytoP (PPA_1_-II), after alkaline hydrolysis 16-B_1t_-PhytoP (PPB_1_-I) and 9-L_1t_-PhytoP (PPB_1_-II) and in preparations without any hydrolysis 16-E_1t_-PhytoP (PPE_1_-I) and 9-D_1t_-PhytoP (PPE_1_-II), not in a free form, but bound to glycerol (Gro). Since it is known that 16-E_1t_-PhytoP (PPE_1_-I) undergoes a dehydration to16-A_1t_-PhytoP (PPA_1_-I) and 16-B_1t_-PhytoP (PPB_1_-I); and 9-D_1t_-PhytoP (PPE_1_-II) to 9-J_1t_-PhytoP (PPA_1_-II) and 9-L_1t_-PhytoP (PPB_1_-II), we speculated that these species originated from16-E_1t_-PhytoP (PPE_1_-I) and 9-D_1t_-PhytoP (PPE_1_-II).

Fractionation on silica column yielded 7 fractions, but only in 3 of them, namely fraction 3, 4, and 5 we have detected lipid mediators. A major compound of fraction 3 analyzed after acidic hydrolysis was 16-A_1t_-PhytoP (PPA_1_-I) and 9-J_1t_-PhytoP (PPA_1_-II). After alkaline hydrolysis fraction 3 and 4 (0.028 and 0.048% to APE, respectively) contained 16-B_1t_-PhytoP **(**PPB_1_-I) and 9-L_1t_-PhytoP (PPB_1_-II), whereas fraction 5 (0.12% to APE) 16-F_1t_-PhytoP (PPF_1_-I) and 9-F_1t_-PhytoP (PPF_1_-II). Other detected compounds in various fractions were alkenes, alkanes, free fatty acids, di- and tri-hydroxy fatty acids.

**Figure 1:**
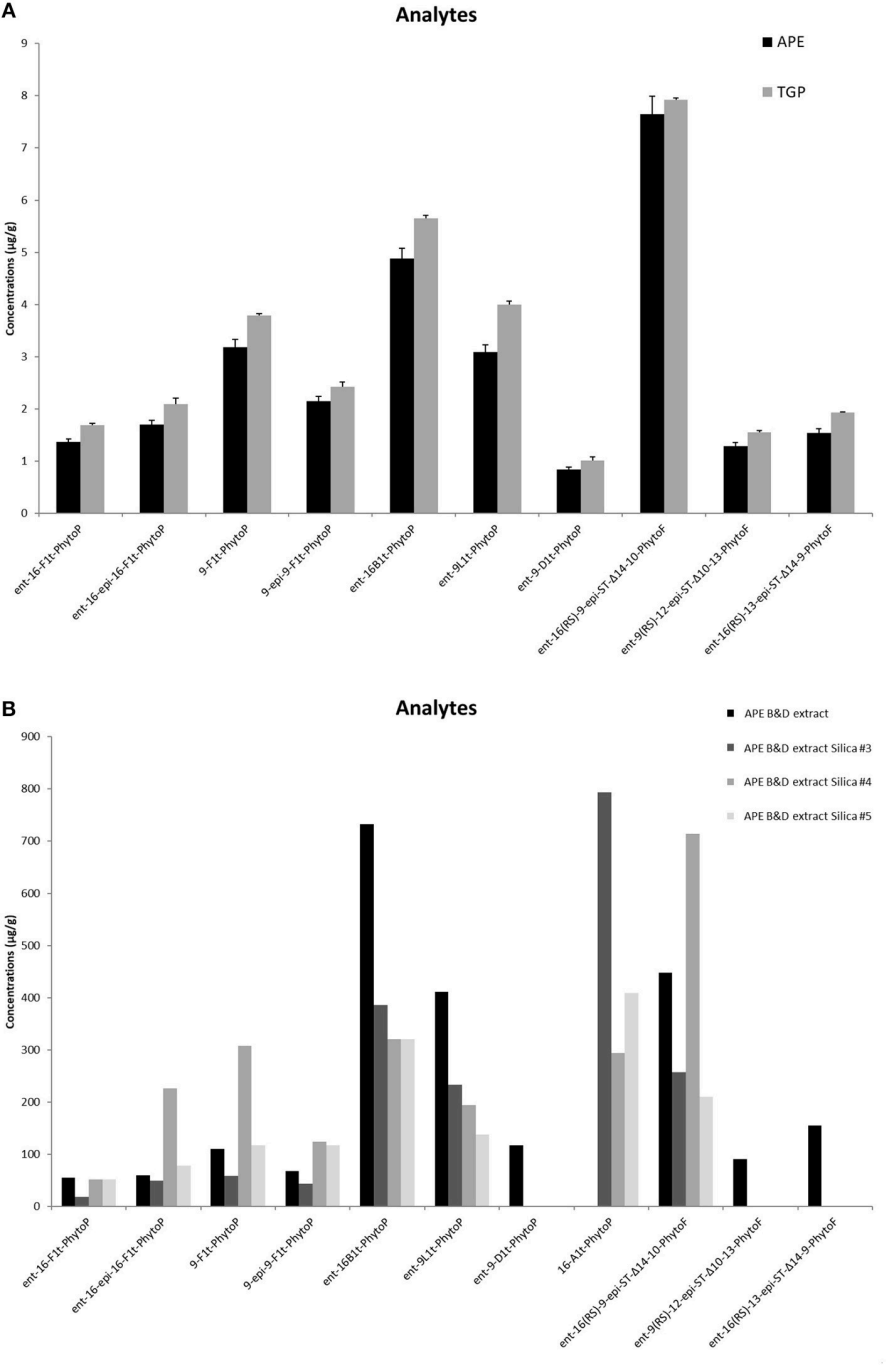
Content of phytoprostanoids in Timothy grass pollen (TGP) and aqueous pollen extract (APE) **(A)**, and in the lipid-enriched APE_B/D_ extract and fractions 3, 4, 5 obtained after silica gel fractionation of APE_B/D_ **(B)** measured in LC/MS method. The content is given as mg/g of the sample. Quantification was achieved by the ratio between the peak area of each analyte and that of the corresponding internal standard.

Qualitative and quantitative LC/MS analysis of APE revealed the presence of PhytoPs from F_1t_-, B_1t_-, L_1t_-, D_1t_-series but also PhytoFs detected for the first time in pollen extracts, that would be considered to be new metabolites due to their recent discovery in nuts, seed, melon leaves or macroalgae (30). Importantly, the amounts of PhytoPs and PhytoFs detected in APE were comparable to those present in whole grass pollen (Figure 1A). We have enriched the lipid fraction of APE performing Bligh/Dyer (APE_B/D_) extraction that led to the increase in detected lipids in factor of around 100 (Figure 1B). For a quantitative point of view, *ent*-16-B_1t_-PhytoP (PPB_1_-I) constituted the most abundant with a yield of 730 μg/g of pollen, while the lowest content was 55 μg/g of *ent*-16-F_1t_-PhytoP (PPF_1_-I). The content of PhytoFs reached values of 448, 155, and 90 μg/g for *ent*-16(*RS*)-9-*epi*-ST-Δ^14^-10-PhytoF *ent*-16(*RS*)-13-epi-ST-Δ^14^-9-PhytoF and *ent*-9(RS)-12-*epi*-ST-Δ^10^-13-PhytoF, respectively. The separation of APE_B/D_ on silica gel produced 3 fractions that presented specific profiles in terms of quality and quantity. Indeed, compared to APE_B/D_ deprived of PhytoPs from A-serie, sub-fractions contained large amounts of 16-A_1t_-phytoP, ranging from 293 to 793 μg/g. In contrast, *ent*-16(RS)-13-epi-ST-Δ^14^-9-PhytoF and *ent*-9(RS)-12-*epi*-ST-Δ^10^-13-PhytoF have been identified only in APE_B/D_. If we compare the 3 sub-fractions to each other, the purification process allowed the concentration of 16-A_1t_-PhytoP in fraction 3, while faction 4 concentrated in particular *ent*-16-*epi*-16-F_1t_-phytoP, 9-F_1t_-phytoP and *ent*-16(RS)-9-*epi*-ST-Δ^14^-10-PhytoF.

### 16-E_1t_-PhytoP (PPE_1_-I) and 9-D_1t_-PhytoP (PPE_1_-II) Are Bound to Glycerol

**Figure 2:**
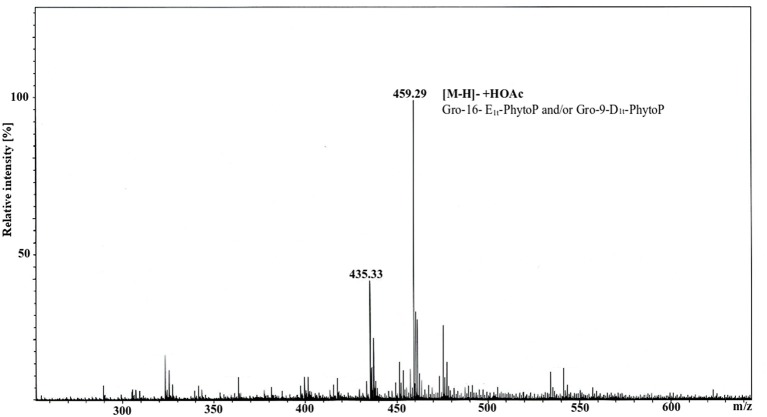
ESI MS spectrum of fraction 3 originating from silica gel separation of APE_B/D_. The measurement was performed in negative ion mode using an amaZon speed ETD. Molecular ion 459.29 (calculated 459.26) corresponded to [M-H]^−^ +HOAc consisting of Gro-16E_1t_-PhytoP/Gro-9-D_1t_-PhytoP.

GC/MS analysis of APE_B/D_ performed without any hydrolysis provided evidence that 16-E_1t_-PhytoP (PPE_1_-I) and 9-D_1t_-PhytoP (PPE_1_-II) are present not in a free form but bound to Gro. To further prove this, we run ESI MS analysis of fraction 3, and indeed the highest molecular ion 459.29 m/z corresponded to Gro-PhytoP-E_1_ (Figure 2). As in GC/MS we have found both stereoisomers of PhytoP-E_1_, namely 16-E_1t_-PhytoP and 9-D_1t_-PhytoP, both of them can be present in APE bound to Gro. We attempted to enrich this compound, and purified fraction 3 further on HPLC. This led to the isolation of Gro-16-E_1t_-PhytoP and Gro-9-D_1t_-PhytoP as determined by GC/MS (data not shown).

### APE Recruits Mast Cells, Induces Their Activation, and Enhances Degranulation

**Figure 3:**
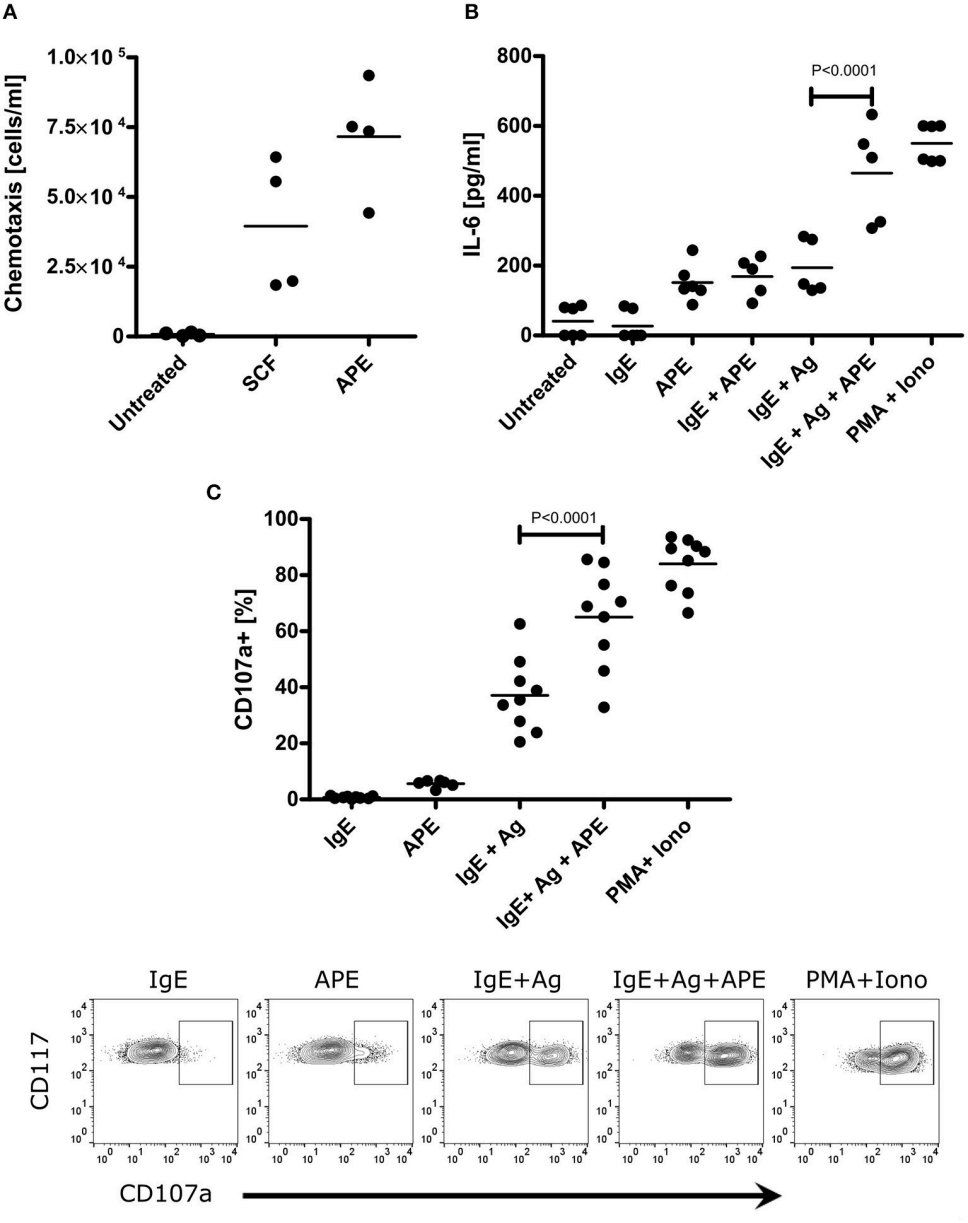
APE induces BMMC chemotaxis, IL-6 production and enhances IgE/Ag-mediated effects. **(A)** BMMCs migrated through the polycarbonate filters toward APE. Assay buffer was used a negative control (marked as untreated), SCF was used as positive control. **(B)** APE induces IL-6 release in unsensitized BMMCs and enhances its production upon stimulation with IgE/Ag. BMMCs generated from C57BL6 mice were left untreated, stimulated with APE, DNP-HSA with or without IgE-sensitization. PMA and Ionomycin were used as positive control. IL-6 release into the supernatant was measured by ELISA. Data from two independent experiments (*n* = 5–6) are shown. **(C)** Incubation with APE induces BMMC degranulation (in average 6%) in IgE/Ag-independent manner and strongly enhance IgE/Ag-induced degranulation measured by CD107a translocation to plasma membrane. Incubation of BMMCs only in the presence of IgE was used as negative control and stimulation with PMA/Ionomycin was used as positive control. Cells were stained for CD117, FcεRIα and CD107a expression, and acquired on a BD FACScalibur flow cytometer. Bullet points represent the frequencies of FcεRIα^+^CD117^+^CD107a^+^ (degranulated) BMMCs. Representative density plots (**C**, down) show how each of the treatments influence the degree of mast cell degranulation. Depicted are results out of 3 experiments generated from independent BMMC cultures (*n* = 9). Means ± SD are shown as lines. Statistical significance was calculated using the 1-way ANOVA analysis followed by Bonferroni's post-test for selected pairs of columns (IgE+Ag vs. IgE+Ag+APE).

Mast cells (MCs) are well-recognized as essential effector cells during allergic reactions, where their recruitment to the site of insult is critical (31). Hence, we were curious whether APE would induce chemotaxis of mast cells and influence their activation. BMMCs strongly migrated toward APE (Figure 3A), indicating the presence of chemotactic to mast cells compounds. This effect was even stronger than that of SCF, a known chemotactic factor for mast cells. MCs exert major effector functions by rapid degranulation and release of a wide range of mediators upon IgE-mediated FcεR crosslinking. BMMCs were treated with APE alone or during the induction of degranulation by sensitization with dinitrophenol (DNP)-specific IgE (IgE) followed by incubation with DNP-human albumin (Ag). Importantly, APE led to BMMCs degranulation in an IgE/Ag independent manner (Figure 3B) and significantly increased IgE-mediated degranulation of BMMCs. The same results we have obtained for protein-free APE (APE_ProtK_) (not shown). Functionally, APE induced production of IL-6 (Figure 3C) in unsensitized BMMCs and enhanced IL-6 production in cells stimulated with IgE/Ag, overall indicating that APE has a capacity to recruit mast cells, activate them and induce their degranulation.

### APE Selectively Induces CD1d Upregulation on Dendritic Cells

**Figure 4:**
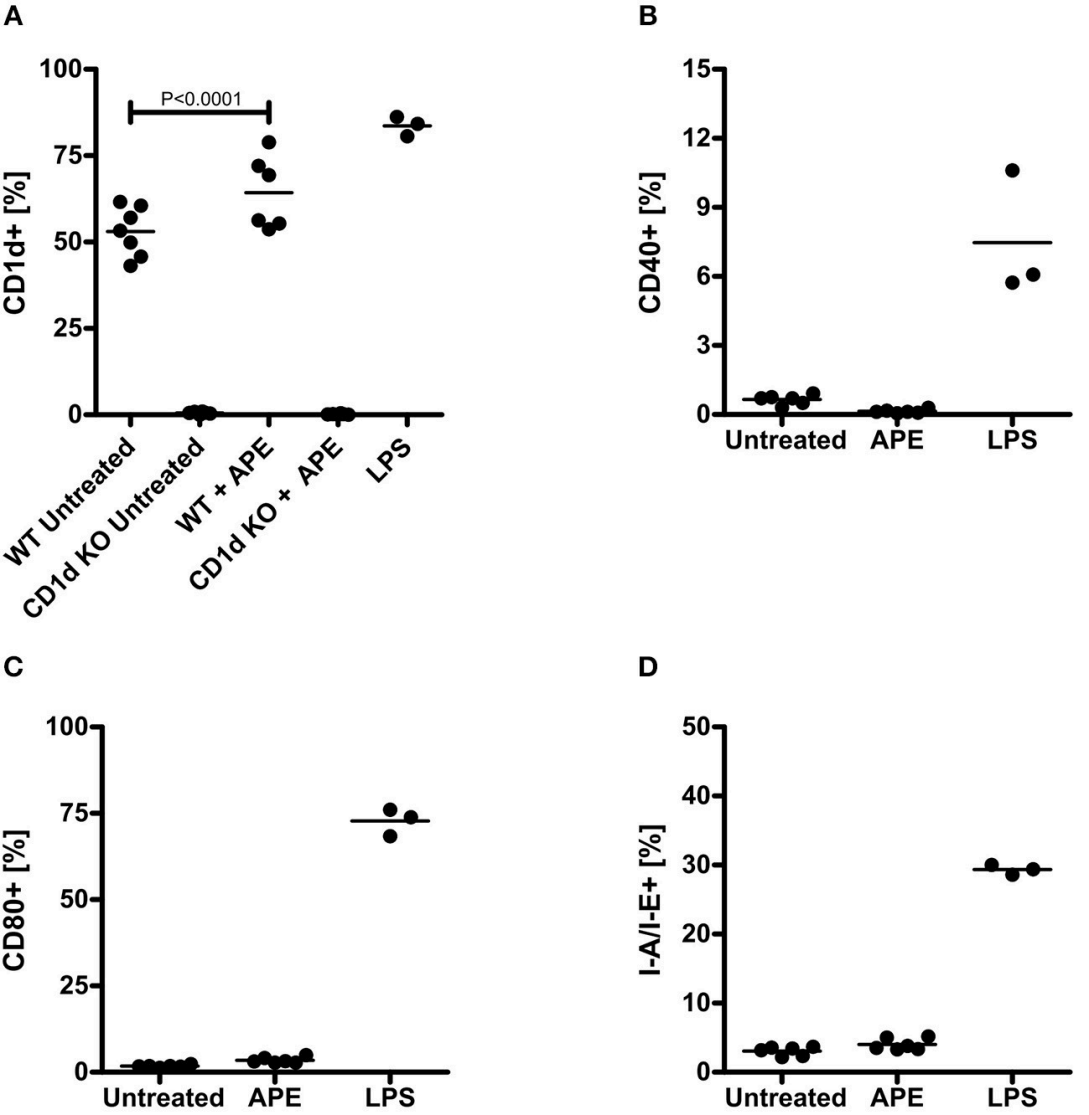
Activation of dendritic cells by APE. APE selectively induced expression of CD1d **(A)** but not of CD40, CD80 and MHC class II on BMDCs **(B–D)**. Negative control BMDCs were left unstimulated (marked as untreated) and positive control BMMC were activated with LPS. Cells were stained for CD1d, CD40, CD80, and MHC-class II expression and analyzed on a BD FACScalibur flow cytometer. Bullet points represent the percentage of cells expressing the corresponding activation markers. Graph shows combined results out of 2 experiments generated from independent BMDC cultures (*n* = 3–7). Means ± SD are shown as lines. Statistical significance was calculated using the 1-way ANOVA analysis followed by Bonferroni's Multiple Comparison post-test.

Expression of co-stimulatory molecules on dendritic cells influence further antigen recognition and polarization of T cells. To gain more understanding on the mechanisms how APE modulates immune responses and to evaluate whether APE could generally regulate recognition of other lipid classes such as glycolipids, we analyzed changes in the expression of CD1d on BMDCs in response to treatment with APE. *Cd1d*^−/−^ BMDCs were used to rule out non-specific CD1d staining. Surface expression of CD1d on BMDC was significantly increased in APE stimulated BMDCs (Figure 4A). Contrary to LPS, known to induce expression of CD40, CD80, and MHC-class II, APE had no influence on the expression of these co-stimulatory molecules (Figures 4B–D).

### Glycerol-Bound PhytoPs Mirror the Effect of APE on the BMMC Degranulation

**Figure 5:**
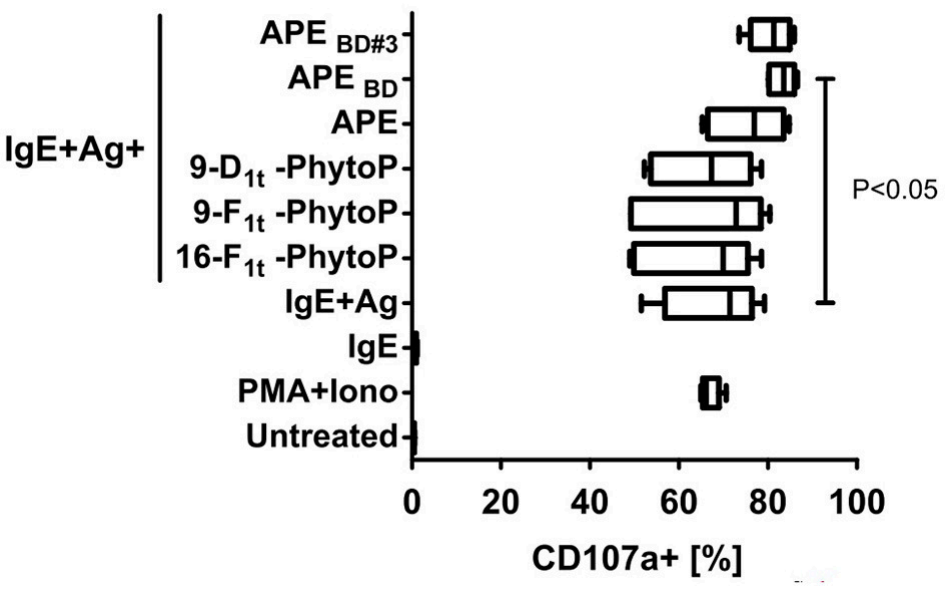
Glycerol-bound phytoprostanes are responsible for the enhancement of MC degranulation. APE_B/D_ (containing enriched Gro-phytoprostanes) and enriched Gro-16E_1t_-PhytoP/Gro-9-D_1t_-PhytoP (APE_B/D#3_) fraction but not free PhytoP led to enhanced IgE/Ag-induced degranulation of BMMCs as measured by CD107a translocation to plasma membrane. Negative control BMMCs were left unstimulated (marked as untreated) and positive control BMMC were activated with PMA and Ionomycin. Cells were stained for CD117 and CD107a and acquired on a MACSQuant10 flow cytometer. Box & whiskers plots represent the frequencies of degranulated (CD107a^+^) BMMCs (CD117^+^ events). Graph shows combined results out of 2 experiments generated from independent BMMC cultures (*n* = 6). Means ± SD are shown as lines. Statistical significance was calculated by One-way ANOVA followed by Dunnett's multiple comparison post-test to a control group (IgE+Ag).

Previous reports questioned the role of PhytoP in Th2 polarizing capacity of APE (14). Since our chemical analyses revealed that PhytoP in grass pollen are mainly present in a bound and not free form, we were prompted to test, whether the effect of APE on the IgE/Ag dependent degranulation of BMMCs would be reproduced by free or isolated by us fractions enriched with 16-E_1t_-PhytoP and 9-D_1t_-PhytoP. BMBCs were sensitized with IgE specific for DNP-human albumin (IgE) followed by incubation with DNP-human albumin (Ag) and co-incubated either with synthetic free PhytoP (16-F_1t_-PhytoP (PPF_1_-I), 9-F_1t_-PhytoP (PPF_1_-II), 9-D_1t_-PhytoP (PPE_1_-II), or APE, APE_B/D_, APE_B/D#3_ (possessing as major compounds 16-E_1t_-PhytoP, PPE_1_-I and 9-D_1t_-PhytoP, PPE_1_-II). Importantly, only APE, APE_B/D_, APE_B/D#3_ and not free PhytoP led to increased IgE/Ag dependent degranulation of BMMCs, indicating that biological activity of APE might be mediated by bound-PhytoP (Figure 5). The effect observed for APE_B/D_ was statistically significant.

## Discussion

It has been demonstrated that pollen in addition to liberating protein allergens rapidly release various bioactive lipids into the aqueous phase (32, 33). These pollen-associated lipid mediators (PALMs) were shown to stimulate and recruit cells of the innate immune system, such as neutrophils (7) and eosinophils (13). Furthermore, PALMs influenced the activation and functional maturation of human DCs toward a Th2 type (12). APE, the protein-free fraction of ragweed pollen extracts (Amb-APE), and the pollen-derived PhytoP-E_1_ were shown to be responsible for B-cell-dependent aggravation of IgE-mediated allergies (34). In this report the stereoisomer of PhytoP-E_1_ was not further specified.

LC/MS analysis of APE and APE_B/D_ revealed that the content of phytoprostanoids (μg/g) was enriched in APE_B/D_ by a factor of 100. In comparison with the previous reports (12) we have determined specifically the stereoisomers of PhytoP found in grass pollen, e.g., 16-E_1t_-PhytoP and to our knowledge for the first time reported the presence of PhytoF. PhytoFs are tetrahydrofuran ring containing compounds created as PhytoP from α-linolenic acid (ALA,18:3 n-3) and are proposed to be indicators of oxidative stress in plants, similarly to PhytoPs (30). Since the content of PhytoF was even higher than those of PhytoP it would be interesting to evaluate the role of synthetic PhytoF in the context of allergy.

Our analyses did not show any presence of hydroxy fatty acids-derivatives of linoleic acids (HODEs) in Timothy grass pollen contrary to work of Traidl-Hoffmann et al. (7). Along with the work of Imbusch and Mueller indicating that levels of esterified PhytoP-F_1_ and PhytoP-E_1_ were one to two orders of magnitude higher than those of free PhytoPs (35) we have found that 16-E_1t_-PhytoP (PPE_1_-I) and 9-D_1t_-PhytoP (PPE_1_-II) were bound to glycerol. Since we did not detect any 16-F_1t_-PhytoP (PPF_1_-I) and 9-F_1t_-PhytoP (PPF_1_-II) in the sample without hydrolysis, and in the samples after hydrolysis first after a fractionation step, we speculate that these PhytoP species are also esterified, having a molecular weight too high for the limit detection in GC/MS.

Definitively the method of choice for the detailed qualitative and quantitative analyses of prostanoids is LC/MS. This is a very sensitive method and due to application of internal standards (synthetic neuroprostane (36)) the identification of different isomers is achieved. The limitation of LC/MS is the detection of artifacts originating from products of alkaline hydrolysis and incapability of measuring bound (esterified) molecules.

The disadvantage of GC/MS is the requirement of a derivatizing process and the inability to separate isomeric compounds with the same molecular weight. Fortunately, we have succeeded in performing GC/MS analyses on the sample without hydrolysis and by doing so detected 16-E_1t_-PhytoP (PPE_1_-I) and 9-D_1t_-PhytoP (PPE_1_-II) bound to glycerol. This finding we have reproduced in ESI MS experiment of fraction 3. Thus, the combination of the analytical protocols is of great value to understand the chemical content of biological samples. The fact that 16-E_1t_-PhytoP (PPE_1_-I) and 9-D_1t_-PhytoP (PPE_1_-II) (we speculate that this is also the case for 16-F_1t_-PhytoP (PPF_1_-I) and 9-F_1t_-PhytoP (PPF_1_-II)) is not present in free but bound form, may explain why E_1_- and F_1_-PhytoPs did not display Th2-polarizing capacities *in vivo* (14) as has been shown in *in vitro* studies (12). Our results showing that APE, APE_B/D_, APE_B/D#3_ and not free PhytoP led to an increased IgE/Ag dependent degranulation of BMMCs support this hypothesis. It is also tempting to speculate that PhytoFs, found for the first time in grass pollen may be another important player in APE.

Mast cells are strategically located at sites that are continuously exposed to the environment, such as mucosal surfaces, and together with their immediate effector activity, they are considered as sentinels of the immune system. In line with this concept, the notion that lipid mediators are immediately released upon contact with the mucosal surfaces, they potentially recruit or induce an accumulation of mast cells to the site of release as evidenced by their recruitment by PALMs. Mast cells are thus the third type of effector cells of allergic reaction, along with neutrophils and eosinophils to which PALMs of pollen grass act chemotactic (7, 13).

In addition, dendritic cells would be in turn also recruited and activated by PALMs. During the process of maturation, dendritic cells migrate to the regional lymph node and prepare for antigen presentation. Interestingly, PALMs induced a selective upregulation of CD1d, probably by activation through PPARγ ligation (37, 38), which could indicate that PALMs induces a maturation program that prepares DCs for glycolipid presentation of potential glycolipid ligands co-delivered with PALMs to mucosal surfaces. Further analyses are required to assess the relevance and significance of this pathway for the process of allergic reactions.

This is the first report evidencing PALMs induced upregulation of CD1d. The work of Abós-Gracia et al. (38) demonstrated that the exposure to total lipid extract but not APE of olive pollen to DCs upregulated Cd1d expression that led to the activation of invariant Natural Killer cells (iNKT). Interestingly, this phenomenon was in accordance with our results not linked to the increased expression of other maturation markers, such as CD80 and CD86.

In summary, our work clearly presented that the chemical composition of fast-released lipids in grass pollen is much more complex than evidenced before. Moreover, the function of molecules originating from heterogenous biological systems, such as sources of allergens, has to be synchronized with the knowledge of detailed chemical structure. Functionally, with our work and others, it became clear that PALMs contribute to the initiation and effector phase of allergy, having the capacity to attract/activate major players of allergic inflammation (neutrophils, eosinophils, mast cells). We have additionally evidenced that PALMs may prime the immune system for further recognition of glycolipids by NKT cells through the promotion of the expression of CD1d molecules.

## Ethics Statement

Mouse care and removal of organs was performed in accordance with institutional (Research Center Borstel) guidelines. The full ethics comittee approval was not required, according to the local and national guidelines.

## Author Contributions

NG and KD contributed to the conception and design of the study and interpretation of the data. NG performed dendritic maturation test and the statistical analysis. SD performed mast cell degranulation test. ZO and FK performed mast cell degranulation test, chemotaxis and measured IL-6 production. RE prepared and fractionated APE and APE_B/D_ extracts, performed GC/MS analyses. KJ performed ESI MS analyses. J-MG and TD prepared synthetic phytoprostanes. CV and CO performed LC/MS analyses. KD wrote the first draft of the manuscript. UJ contributed to the interpretation of the data and critically revised the manuscript. NG, KD, and CV prepared figures. KD, NG, CV, and TD wrote sections of the manuscript. All authors contributed to the manuscript revision, read and approved the submitted version.

### Conflict of Interest Statement

The authors declare that the research was conducted in the absence of any commercial or financial relationships that could be construed as a potential conflict of interest.
